# Blockchain enabled data security in vehicular networks

**DOI:** 10.1038/s41598-023-31442-w

**Published:** 2023-03-17

**Authors:** Naseem us Sehar, Osman Khalid, Imran Ali Khan, Faisal Rehman, Muhammad A. B. Fayyaz, Ali R. Ansari, Raheel Nawaz

**Affiliations:** 1 COMSATS University Islamabad, Abbottabad Campus, Abbottabad, Pakistan; 2 OTEHM, Manchester Metropolitan University, Manchester, UK; 3 Department of Mathematics and Natural Sciences, Gulf University for Science and Technology, Mubarak Al-Abdullah, Kuwait; 4 Pro Vice Chancellor, Staffordshire University, Stoke-On-Trent, UK

**Keywords:** Electrical and electronic engineering, Energy infrastructure

## Abstract

Recently, researchers have applied blockchain technology in vehicular networks to take benefit of its security features, such as confidentiality, authenticity, immutability, integrity, and non-repudiation. The resource-intensive nature of the blockchain consensus algorithm makes it a challenge to integrate it with vehicular networks due to the time-sensitive message dissemination requirements. Moreover, most of the researchers have used the Proof-of-Work consensus algorithm, or its variant to add a block to a blockchain, which is a highly resource-intensive process with greater latency. In this paper, we propose a consensus algorithm for vehicular networks named as Vehicular network Based Consensus Algorithm (VBCA) to ensure data security across the network using blockchain that maintains a secured pool of confirmed messages exchanged in the network. The proposed scheme, based on a consortium blockchain, reduces average transaction latency, and increases the number of confirmed transactions in a decentralized manner, without compromising the integrity and security of data. The simulation results show improved performance in terms of confirmed transactions, transaction latency, number of blocks, and block creation time.

## Introduction

Intelligent Transportation System (ITS) allows vehicular networks to enable flexible communication between Vehicle to Vehicle (V2V) and Vehicle to Infrastructure (V2I)^1^^,^^2^. Numerous applications have been proposed to enable vehicles to share real-time and non-real content^3^. The real-time applications may include event data, sensory information, and multi-media streams. Whereas non-real-time data consists of time-insensitive messages, web browsing, and file transfers. Despite improvements in technology, vehicular networks still face numerous challenges. According to World Health Organization (WHO), 1.35 million people die every year due to road accidents. Advancements in automobile communication technologies have boosted the growth of ITS and the Internet of Things (IoT) to address various issues, such as road and rail hazards, traffic congestion, secure authentication, and message dissemination over the network^4–7^.

Recent years have seen Autonomous Vehicles (AVs) as an evolving technology of ITS that has gained significant attention. These vehicles are usually fitted with various kinds of onboard resources, such as sensors, radars, cameras, storage devices, event recorders, etc., to perform different actions, e.g., object detection, congestion monitoring, path finding, and so on^8,9^. The AVs are expected to capture large volumes of data to analyze it and make real-time intelligent decisions in response to the surrounding events. For instance, the sensors fitted with AVs capture gigabytes of data that need to be processed with complex machine learning algorithms to deduce logical results. To achieve communication efficiency, storage, and high-end processing, 5/6G technologies and Road Side Units (RSUs) connected with Mobile Edge Computing (MEC) servers can be leveraged^10^. RSUs receive all the data sent by the vehicles where MEC servers run machine learning techniques to generate useful predictions^11^.

In ITS, different nodes work together and share data. For instance, RSUs generate alerts in case of any unusual incident so that the vehicles approaching from a far distance are informed in advance to avoid any accidents. However, it is important to ensure data privacy so that malicious users may not modify the message, mislead the network, or generate false alarms resulting in road fatalities^12,13^. In the past few years, numerous efforts have been made to develop services to ensure safety, security, and navigation among vehicles. Recently, the blockchain has emerged as a promising technology to address the security issues in centralized systems^14^. Blockchain is a decentralized peer-to-peer data structure that allows records to be stored in a ledger in a manner that cannot be altered^15^. Due to its decentralized nature, blockchain technology has eliminated the need for a central authority or any form of intermediaries. The majority of the nodes make the decision to add data in the form of a block to the blockchain, rather than relying on a centralized controller resulting into single point of failure^15^. Blockchain technology keeps track of all types of transactions made in a network in a provably secure manner. Blockchain has a disruptive impact on various industries including the manufacturing sector, banking, healthcare, finance, and transportation.

With the recent applications in vehicular networks, blockchain technology can enable a smart transportation system that is decentralized, trusted, and secure without any intermediary^12^. The AVs can take full benefit of blockchain that ensures strong interconnections, and execution of smart contract-enabled transactions for certain events. Moreover, V2I communication is a potential solution to enable cooperative intelligence of AVs contributing to improving services and travelling. Several recent works have explored the use of blockchain technology in vehicular networks. The authors in^16^ and^17^ proposed a secure scheme for inter-vehicular communication using blockchain. Kang et al*.*^18^ presented a blockchain-based peer-to-peer electricity trading system known as PETCON to improve secure electricity trading among Plug-in Hybrid Electric Vehicles (PHEVs). Huang et al*.*^19^ proposed a blockchain-based trading model called Lightning Network and Smart Contract (LNSC) consisting of scheduling, registration, authentication, and charging phases. It also stores the transaction data between electric vehicles and charging stations on the blockchain network while using smart contracts to facilitate an automatic trading process. Cebe et al*.*^20^ discussed how the blockchain can be applied in the post-accident investigation process. Their work provides a mechanism using which the data can be stored and shared only with the party conducting the accident investigation.

The use of blockchain technology in vehicular networks helps in improving the security and authenticity of data. However, the conventional consensus algorithms can cause unavoidable delays and excessive computations in block confirmation due to which timely message dissemination becomes difficult and throughput is reduced. To address these issues, we propose a Vehicular network Based Consensus Algorithm (VBCA), to secure the data shared within a vehicular network using a consortium blockchain. Our model is inspired by the Raft consensus mechanism presented in^21^, however, we performed certain customizations that make the proposed algorithm different from the original Raft algorithm. In the Raft algorithm, the voting process takes place for leader election and all the follower nodes are the potential candidates. A follower may become a candidate node for leader election process after random election time out. The candidate votes itself and send request to other followers for voting. If followers have not voted in the current term they vote ‘yes’, otherwise they vote ‘no’. Once the majority of the followers say ‘yes’, the candidate becomes the leader. Other candidates revert to followers. The candidates get votes in a first come first serve fashion. A leader can serve for two terms. On the contrary, in the proposed algorithm, the leader election process begins with the selection of random list of candidate nodes, on the condition that a candidate node should not have remained a candidate node in the previous term. This makes our candidate selection process different from the Raft. After the candidate nodes are chosen, the proposed scheme performs the leader selection among the candidate nodes based on a Verifiable Random Function (VRF)^22^, which is different in the Raft where the majority voting is utilized. However, the voting process has to be repeated in Raft in case of split decision. In addition, the proposed scheme separates the process of transaction confirmation and block confirmation that helps to increase the decentralization of the system. By separating these processes, it may be more difficult for a single entity to control the confirmation of transactions or blocks, thus increasing the overall security and reliability of the system.

Vehicles being part of a public blockchain network can send and receive data (transactions) via the nearest RSU, whereas the RSU acting as a *leader* in a private blockchain verifies and appends the set of transactions in the form of a block to the blockchain ledger. The leader RSU is selected securely using the smart contract in the proposed consensus algorithm. To timely send the message and to increase the throughput over the network, the transaction pool stores the latest transactions that are waiting to be appended to the blockchain ledger. The main contributions of this paper are summarized as follows:

- We propose a VBCA algorithm using smart contracts to improve the network throughput in a blockchain-based vehicular network.
- The smart contract mechanism is used to ensure decentralization by letting multiple RSUs append a block in a ledger. A leader selection process is also proposed to ensure the decentralization in block creation. This also addresses the selfish mining problem.
- We have also reduced the transaction latency by proposing the separation of transaction confirmation and block creation process.
- The proposed technique demonstrates improvement in results when compared with the state-of-the-art mechanisms in terms of throughput and the number of blocks created per node in the vehicular network while ensuring decentralization.

The rest of the paper is organized as follows. "Related work" section discusses the related work. The system architecture is explained in "System architecture" section. The proposed consensus algorithm is discussed in "Security analysis" section. Achievements, limitations, and future research directions evaluates the proposed model. Finally, "Conclusion" section concludes the paper.

## Related work

In recent years, blockchain technology has been applied in numerous application domains, such as product lifestyle management^23^, smart tracking and tracing^24^, and smart transportation, to name a few. Blockchain application on vehicular networks is relatively a new area of research and few works exist in that direction. In the existing literature, there is no standard categorization of blockchain based schemes for vehicular networks. In this section, we present some of the recent works on the application of blockchain for vehicular networks. We categorize the works based on the type of blockchain, consensus algorithms, and applications. Later, at the end of this section we present a comparative summary of the literature in Table 1.

**Table 1:** Comparative analysis of Blockchain in Vehicular networks.

Ref	Problem	Consensus algorithm	Mining Nodes	Limitations
^25^	Secure message dissemination in local Blockchain	Proof of work	Vehicles	Scalability, latency, and minimal number of transactions
^28^	Trust establishment based on reputation mechanism	Proof of work and Proof of Storage	RSUs	High block propagation delay, latency, and require high computational resources
^32^	Cache content based on priority to reduce latency	Practical byzantine fault tolerance	RSUs	Prone to selfish mining attack and high bandwidth consumption
^41^	Trust establishment and reputation scheme for local Blockchain to make data available	Variant of Proof of work	Vehicles	Message control and dissemination
^33^	5G to reduce network latency in Autonomous vehicular network	Byzantine fault tolerance	Vehicles and RSUs	Authentication establishment and security implementation
^40^	Data exchange control and trust management to share messages	Proof of event	RSUs	Security and scalability
^42^	Secure message exchange between mobile nodes using Blockchain and reduced storage overhead	Distributed Time Consensus algorithm	RSUs	System becomes more centralized with time, latency and prone to selfish mining attack
^36^	Secure data sharing and incentive mechanism in vehicular computing edge network using Blockchain	Practical byzantine fault tolerance	RSUs	Network is semi decentralized
^37^	Efficient and honest miner selection for consensus from large pool of miner nodes	Proof of Driving	Vehicles	Transaction related data not evaluated to prove the proposed model impact on network parameters
^38^	The secure data sharing and efficient throughput algorithm is proposed	Score Group practical byzantine fault tolerance	RSUs	The primary node makes the network more centralized

### Public blockchain based solutions in vehicular networks

Shrestha et al*.*^25^ proposed an approach for secure message dissemination among vehicles using a local blockchain. The trust weight of every message and vehicle is stored as a transaction in the blockchain using the Proof of Work (PoW) consensus algorithm. The cars equipped with the highest computational power participated in the mining process. The RSUs were responsible for authentication and providing location certificates to the vehicles within their communication range. The paper proposed the maintenance of a separate blockchain for each region to increase scalability. However, the paper did not address the block propagation delay that occurs due to the increase in the number of vehicles in a local blockchain. Moreover, the PoW algorithm used for mining can only process seven transactions per second, which are less for delay-sensitive vehicular communications.

Yang et al*.*^26^ developed a model for secure yet reliable communication and trust management system for vehicular networks. The study aims to improve the miner selection presented in^25^ by limiting the number of nodes. If a vehicle sends a message to another vehicle, its rating is computed and submitted to RSU. The RSU ranks the vehicle on the blockchain according to the respective ratings. All RSUs serve as nodes and a consensus is reached to add the block to the chain using a joint PoW and Proof-of-Stake consensus algorithm. The integrity and confidentiality of the proposed model using blockchain are achieved, but privacy is still an issue as vehicle identification numbers serve as a public key identifier. Moreover, the size of the rating packet is greater compared to the message packet resulting in the communication overhead. In^27^, the authors experimented with the NS3 simulator to evaluate the performance of the blockchain network in the presence of a selfish miner. Experimental results depict that the block receive time has increased from 10 to 19 min.

### Consortium blockchain based solutions in vehicular networks

A consortium blockchain is presented in^28^ for secure and efficient data sharing amongst vehicles and RSUs. The use of smart contracts ensured access control on RSUs. In addition, the implementation of a reputation-based mechanism by applying a ‘three weight subjective model’ ensured the integrity of shared data. Proof of storage and PoW consensus algorithms are used to store data securely and to generate valid blocks, respectively. The reputation system proposed in^28^ improves the probability of detecting the misbehavior of vehicles by up to 70%. Compared to the previous work^25^, the proposed scheme^28^ showed better results in terms of security. However, the authors ignored the latency and propagation delay of blocks, which is critical to make the model practical for the real-world scenario. In^29^, the authors aimed to reduce the resource consumption to increase the system utility for the traffic signal control mechanism in consortium blockchain. Performance evaluation of the proposed work indicated a decrease in computational cost with the implementation of a smart contract. However, the use of Elgamal encryption can result in higher latency in the network, not discussed in the evaluations.

### Incentive based consensus algorithms

The study in^30^ implemented delegated proof of stake to work out issues like miner’s voting collusion and double-spending attack. The reputation score of each RSU is evaluated by the trusted authority on a blockchain. The miners with the highest reputation are selected as active miners and the remaining are termed as standby miners. To encourage the standby miners to actively verify the blocks, contract theory^30^ is implemented to distribute the reward amongst the miners. After the addition of each block, the new active miner is selected. However, such an approach can delay the processing of the incoming transactions, thereby making it quite impractical to deploy the system in vehicular networks.

Li et al*.*^31^ proposed a scheme that allows vehicles to forward announcement messages without compromising privacy. The authors introduced a model known as CreditCoin having a consensus algorithm that confirms 100 transactions in an overall of 192 ms to append a block in the blockchain. The CreditCoin exhibited improved performance in terms of throughput and latency compared to the previous works, e.g.,^30^ and^32^. The proposed framework is employed for the post-accident analysis and it did not take into account the mobility issue of vehicles.

### Practical Byzantine Fault Tolerance based consensus algorithms

In^32^, the authors presented a secure data caching scheme that considers a message’s popularity and scope at RSUs using hierarchal blockchains. Two layers in the hierarchal blockchain maintained the ledgers. The paper aims to achieve low latency by using a ‘Practical Byzantine Fault Tolerance’ (PBFT) consensus algorithm on both the proposed layers of blockchain. The information is disseminated in the network according to the message priority. Simulation results indicated improved performance over the conventional blockchain system in terms of system failure rate, overall utility, and low latency. The research presented in^33^ discussed the use of 5G technology with blockchain in vehicular ad hoc networks. The work focused on applying the PBFT consensus algorithm to speed up the transaction confirmation. However, the work lacks the evaluations or performance comparisons of the implemented consensus algorithm. The primary focus of the studies^34^ and^35^ is to protect the privacy of vehicles where RSUs are maintaining the blockchain ledger by using the PBFT consensus algorithm. The researchers in^35^ have proposed a solution using PBFT to ensure a more secure environment for vehicles to establish trust levels.

The study in^36^ proposed a blockchain based model of vehicular edge computing networks (VECNets). The proposed model used PBFT as a consensus mechanism which has high transaction performance compared to^15^. The miners are the set of preselected RSUs which makes the network semi decentralized. The performance of the overall network focused more on the incentive mechanism where a vehicle with good ratings is more trustworthy.

In^37^, the authors presented a variant of the PBFT consensus algorithm known as Proof of Driving (PoD). The fair selection of honest miners from a large pool of miners is proposed based on the service standard scoring method. The performance evaluation of the proposed model has shown the number of nodes being selected but the impact of throughput of the proposed network model is not evaluated.

The research presented in^38^ focuses on secure and efficient data sharing amongst the internet of vehicles by proposing a consensus algorithm Score Grouping-PBFT (SG-PBFT). Mainly the SG-PBFT relies on the basic PBFT consensus mechanism but with the scoring criteria which makes the algorithm efficient and reduces the communication overhead. The throughput of the network has shown better performance. The proposed consensus process is highly dependent on the primary node making it inclined towards centralization.

### Custom consensus algorithms

The branched blockchain concept proposed in the study^39^ is a lightweight decentralized ledger system that easily integrates the recent block with an existing P2P network. To improve the lightweight property, the block maintenance design of this framework estimates the number of active and inactive blocks in the network and maintains only active blocks rather than complete blocks. It enables the network to combine all these active blocks into a single lightweight blockchain, allowing it to be used even by low-powered vehicles and devices at the physical layer. The study misses the details of PDP consensus mechanisms that are applied to the presented model.

Vehicle to vehicle communication and calculation of the trust level of messages received by other vehicles is the main aim of the research in^40^. Proof of Event (PoE) is implemented to accelerate the message dissemination as a two-way process. RSUs collect the messages, compute the score, and broadcast the results to surrounding vehicles. The performance of PoE has been compared with the PoW and Proof of Activity (PoA). The results showed that the synchronization of blocks in PoE is better than in PoW. However, storing the information of all the events can lead to a scalability issue which is not discussed in^40^. Wagner et al*.*^41^ developed a variant of the PoW consensus algorithm for block creation in which the platoon leader selects the miner with more computational power. Simulation results indicated that the system could process 100 transactions in 200 s. However, the PoW requires greater computation time and is inefficient to be deployed for vehicular networks. Moreover, the paper did not illustrate the procedure to re-elect a platoon leader. Furthermore, the information is not managed globally. A selfish miner in a blockchain network causes the other miners to use more computing resources, thereby significantly affecting the system performance.

### Blockchain based solutions for IoT

In^42^, a Lightweight Scalable Blockchain (LSB) has been proposed with the custom consensus algorithm known as a distributed consensus algorithm. The cluster head is responsible for verifying the transaction and storing it in a blockchain ledger after waiting for a specific amount of time. The results compared with Bitcoin^15^ in terms of throughput indicated improved performance in terms of transactions per second. However, the proposed system has a limitation that over time, it moves toward centralized block mining. Table 1 presents a summary of selected schemes discussed in the literature review.

### System architecture

**Figure 1:**
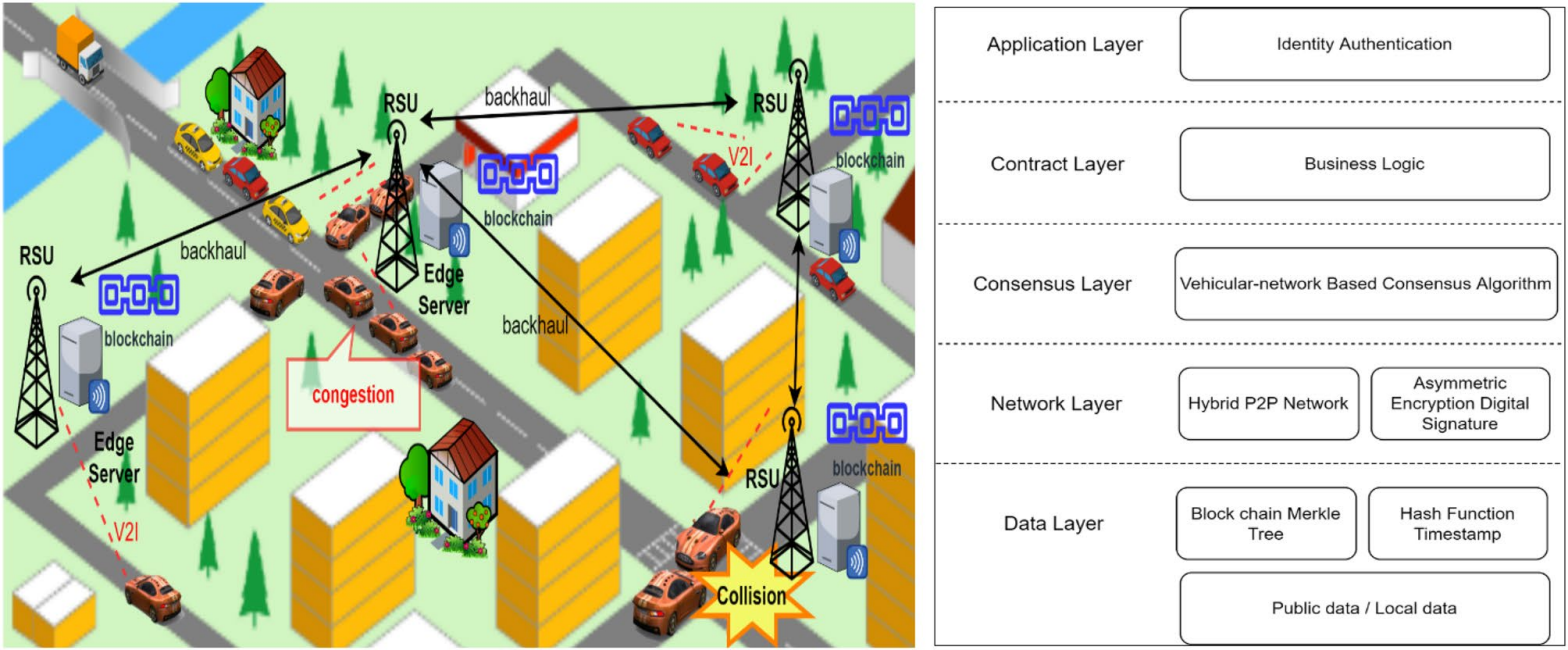
Vehicular blockchain architecture.

This section discusses the major components of the proposed system and the layered architecture. We define two types of nodes as (a) Stationary nodes and (b) Mobile nodes. As shown in Fig. 1, stationary nodes are the RSUs connected with high-power edge servers. Each stationary node is providing geographic coverage to a specific region on the map, and all stationary nodes are interconnected via backhaul links. It is the responsibility of a stationary node to append a block to the blockchain, and a copy of the blockchain is stored by all the stationary nodes in the network^43^. A mobile node captures event data with its sensors, e.g., accidents, congestion, road condition, etc., and sends the data to the nearest stationary node. Due to the P2P network, the vehicles have more reliable connections to the nearby RSUs by using the Dedicated Short Range Communication protocol (DSRC)^1^. Vehicles and RSUs communicate within their specific range using DSRC which reduces the latency between the communicating peers.

The proposed consensus algorithm runs on the edge servers to append the validated transaction in the blockchain stored on the edge servers. Since the vehicles are equipped with different types of sensors, e.g., autonomous vehicles can be equipped with cameras, radars, etc., the large volumes of data are received by the edge servers. Based on the collected data, various statistics and machine learning tools can be applied to train the models to generate various predictions for different applications. For example, by applying predictive learning-based approaches, it would be possible to predict the expected load on various sections of the transportation network during a certain time window. The predicted information can be stored in a separate blockchain and shared among all the stationary nodes through which the vehicles can query the information. The working of the proposed system is divided into following layers.

### Application layer

The application layer facilitates the end users (vehicles) to perform general input/output operations in the system. The user input is passed to the contract layer where the smart contract is called to check the user authorization and data access controls.

### Contract layer

This layer validates the authentication of vehicles and stationary nodes which are interacting with each other via blockchain and deploys the smart contract. The logic embedded in the smart contract once executed cannot be reversed.

### Consensus layer

The consensus algorithm uses mathematical rules to establish trust amongst the nodes in the network. All the stationary nodes that are RSUs connected with servers are maintaining the same copy of the ledger across the network using the consensus algorithm. The RSUs are distributed entities responsible for managing the ledgers of a region for a blockchain thus reducing the network traffic overhead. At present, there are many consensus algorithms of blockchain to achieve a domain-specific objective. In our research, we proposed a custom consensus algorithm discussed in “Security analysis” section.

### Network layer

All nodes in the network are connected in a hybrid P2P manner without any central authority. Every node uses a discovery protocol to find its nearest neighbor RSU to establish links and exchange messages. The key management and cryptographic algorithms that are used to prevent attacks are also part of a network layer.

### Data layer

This layer is incharge of managing the transactions and blocks in the ledger. Hash function, timestamp, and Merkle tree structure^44^ are applied to ensure the integrity and security of the data. The proposed system is based on consortium blockchain having the advantages of both public and private blockchain. The network is public for vehicles to join with the public key pair issued by CA. This is to ensure integrity and authenticity in the vehicular network. The consensus amongst the stationary nodes to maintain the consistent ledger is based on a private blockchain.

### Vehicle registration

Vehicles acquire the public key pair from CA by fulfilling the requirements. CA registers the vehicle and generates the public key pair. On first interaction with the stationary node, the vehicle submits the public key pair with certificates. The stationary node verifies the public key pair from the global certification authority and sends it over the network to store the public key pair.

### Hybrid peer-to-peer network

**Figure 2:**
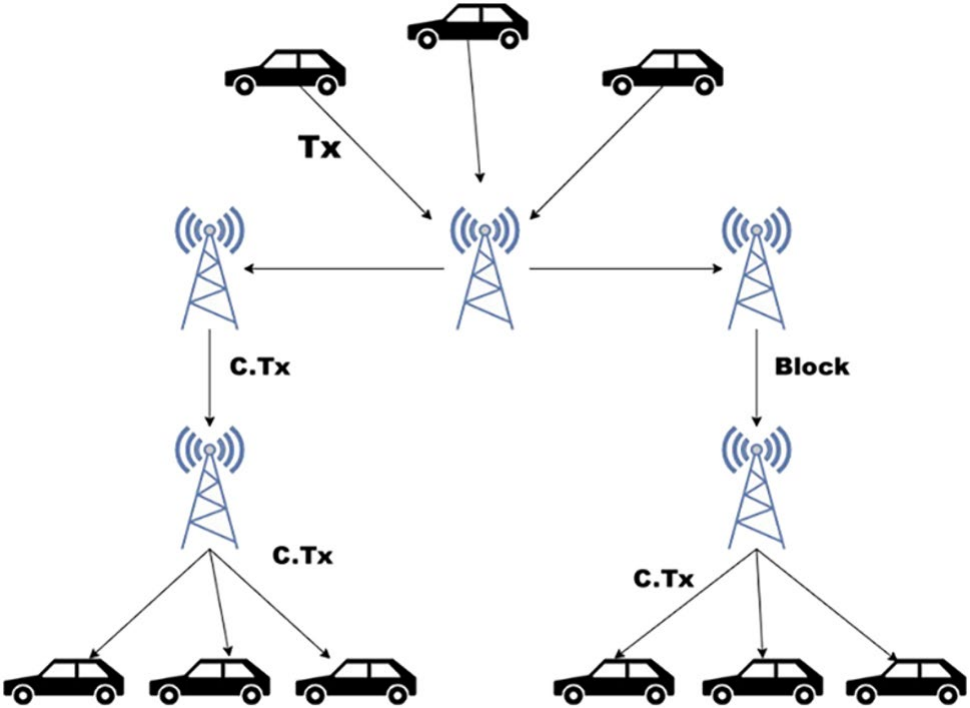
Hybrid peer-to-peer structure.

The proposed network architecture is different from traditional blockchain by being P2P and distributed architecture as shown in Fig. 2. The proposed system is a hybrid P2P making a stationary node a central access point for the vehicles available in its range. Only the transactions that are verified by the leader stationary node are propagated to the other stationary nodes. Finally, the confirmed transactions are broadcasted by the leader to other stationary nodes, and subsequently across the whole network.

### Proposed methodology

First, we present some necessary assumptions as a basis of the proposed methodology:

- Cryptographic primitives (for instance, encryption schemes and hash functions) provide a secure communication channel between entities (vehicle to RSU and RSU to RSU).
- A malicious node is not able to compromise half of the nodes in the network. This is a reasonable assumption in practice.
- RSUs and CA are equipped with hardware having high computation power.

Any information sent by the mobile nodes to stationary nodes is considered as a transaction. The transactions are created by the On-Board Units (OBUs) of the vehicles. All the new transactions are digitally signed by the mobile nodes and propagated to the nearby stationary node. Blockchain technology relies on Public Key Infrastructure (PKI) for transaction verification. As considered by earlier research^25^, we assume that all the vehicles are registered with a Vehicular Authority (VA) and every vehicle and RSU is allotted with the public key pair. A vehicle creates a transaction Tx by encrypting the following: message data, time stamp at which the message was generated, and the hash of the message data. The encrypted transaction is then sent to the stationary node in the range of the respective vehicle. The stationary node uses the public key of the vehicle to decrypt the transaction to get hash value, and then generates the hash of the original received message. If the generated hash value matches with the hash value sent by the vehicle, the transaction is validated. Once validated, the transaction is added into a transaction pool by the leader RSU, as well as simultaneously relayed to other stationary nodes. This ensures the quick spread of information in case of any emergency event.

### VBCA consensus algorithm

This section explains the proposed VBCA algorithm for blockchain based vehicular networks. The consensus algorithm is implemented only at the stationary node level and is further divided into sub-components explained subsequently.

### Leader RSU selection

**Figure 3:**
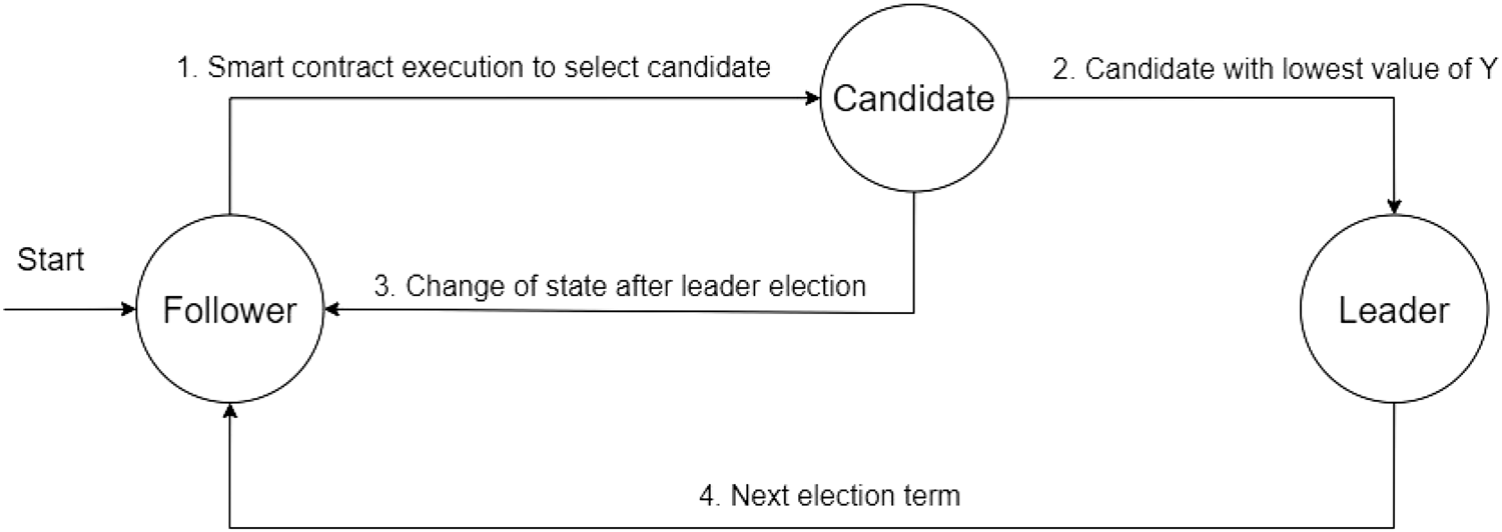
Leader selection process.


As shown in Fig. 3, a stationary node can transition between three states in the consensus process, which are: follower, candidate, or a leader. The stationary nodes are synchronized via a smart contract. The leader selection process is illustrated in Algorithm 1. Initially, a list of all stationary nodes is obtained (Line 1). The list of candidate nodes is selected randomly via using a smart contract among all the follower nodes (Line 2). A selected candidate node must not have remained as a candidate node in the previous term (Line 2). Once the candidate nodes are selected for the leader selection process, the leader is chosen based on a Verifiable Random Function (VRF) ^22^ amongst the candidate nodes. Each candidate has a pair of public and a private key. Given that the value of Q is a seed string and is known to the entire network, the candidate calculates the values (Y, p) = VRF(Pr, Q), where p is the proof of work performed by the node. Each candidate node is required to calculate the value pair (Y, p). The first candidate node that computes the lowest value of Y than a threshold is selected as leader for the current term (Line 3).

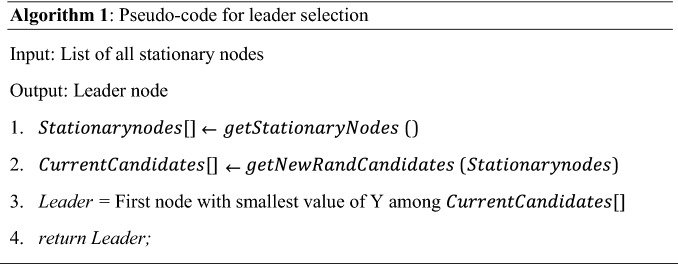


The leader then creates a block and propagates the block over the network of stationary nodes. By using the proof p and public key of the leader node, any candidate node can verify that Y is computed correctly or not.

### Block generation

The block generation takes place after the leader selection. Algorithm 2 shows the block generation process.

- Initializations (Line 1 − Line 2): The transactions that are validated are added to the transaction pool (TxPool), and the added transactions are sorted according to the timestamps.
- In Line 3–Line 6, the sorted transactions are picked by the elected leader of the term. The elected leader picks 10 transactions from transaction pool based on first in first out structure. The transactions, timestamp, hashes of transactions, smallest value of Y, and hash of previous block are then encrypted by using the private key of a leader and the block is broadcasted to the rest of RSU nodes. The follower nodes validate the block by decrypting the block with leader’s public key that is known to all the RSUs node and verify the leadership by verifying the value of Y. After the verification of block, RSU node appends the block into its ledger and sends acknowledgement to the leader. The leader waits for the acknowledgement from the follower nodes according to heartbeat timeout.
- In case the ‘if statement’ of Line 3 is false, the leader selection process in Line 7 executes.

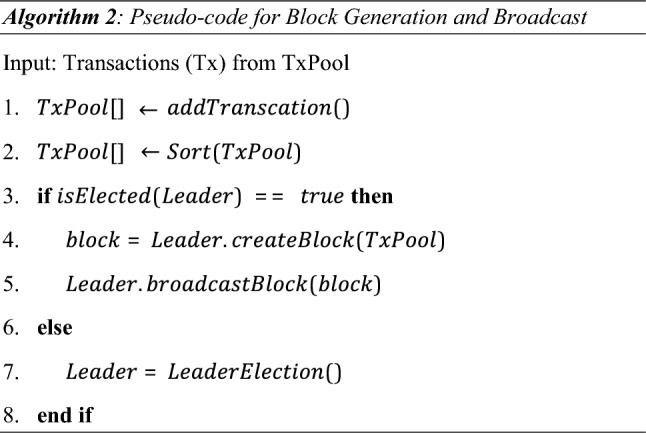


#### Performance evaluation

To the best of our knowledge, there does not exist any special purpose simulator to implement blockchain on vehicular networks. Network Simulator 3 (NS-3) has been widely used for simulating the different types of networks with customized requirements and parameters. Inspired by the study presented in^42^, we found NS-3 an appropriate candidate for our proposed work due to the availability of support libraries addressing the custom requirements of blockchain. We have simulated the proposed VBCA algorithm using smart contracts over a vehicular network. The proposed system is compared with the following consensus algorithms: (a) Distributed Time Consensus (DTC)^42^, (b) Practical Byzantine Fault Tolerance (PBFT)^32^, and (c) PoW^25^. The simulation parameters are discussed in Table 2.

**Table 2:** Simulation parameters and values.

Parameter	Value(s)
Total nodes	50
Vehicles (mobile nodes)	34
RSUs (stationary nodes)	16
Miners	16
Leader	1 (per round)
Candidates for leader selection	4
Genesis block transactions	10
Transaction rate	5000
Transaction size	200 Kb
Block size	2 MB
Heartbeat timeout	50 ms
Total blocks	50

The following performance parameters are used in the comparisons: (a) block confirmation time, (b) throughput, (c) transaction latency, and (d) confirmed transactions. The time from block creation to its confirmation is known as block confirmation time. Throughput is the number of confirmed transactions per second. A large number of transactions are generated over the period, but we are considering only the confirmed transactions in the results.

The average transaction latency for *T* transactions is given as:

1 $$ Avg. \;transaction \;latency = \frac{1}{T}\mathop \sum \limits_{i} Generated \;time_{i} - Confirmation \;time_{i} . $$

### Impact of time on throughput

**Figure 4:**
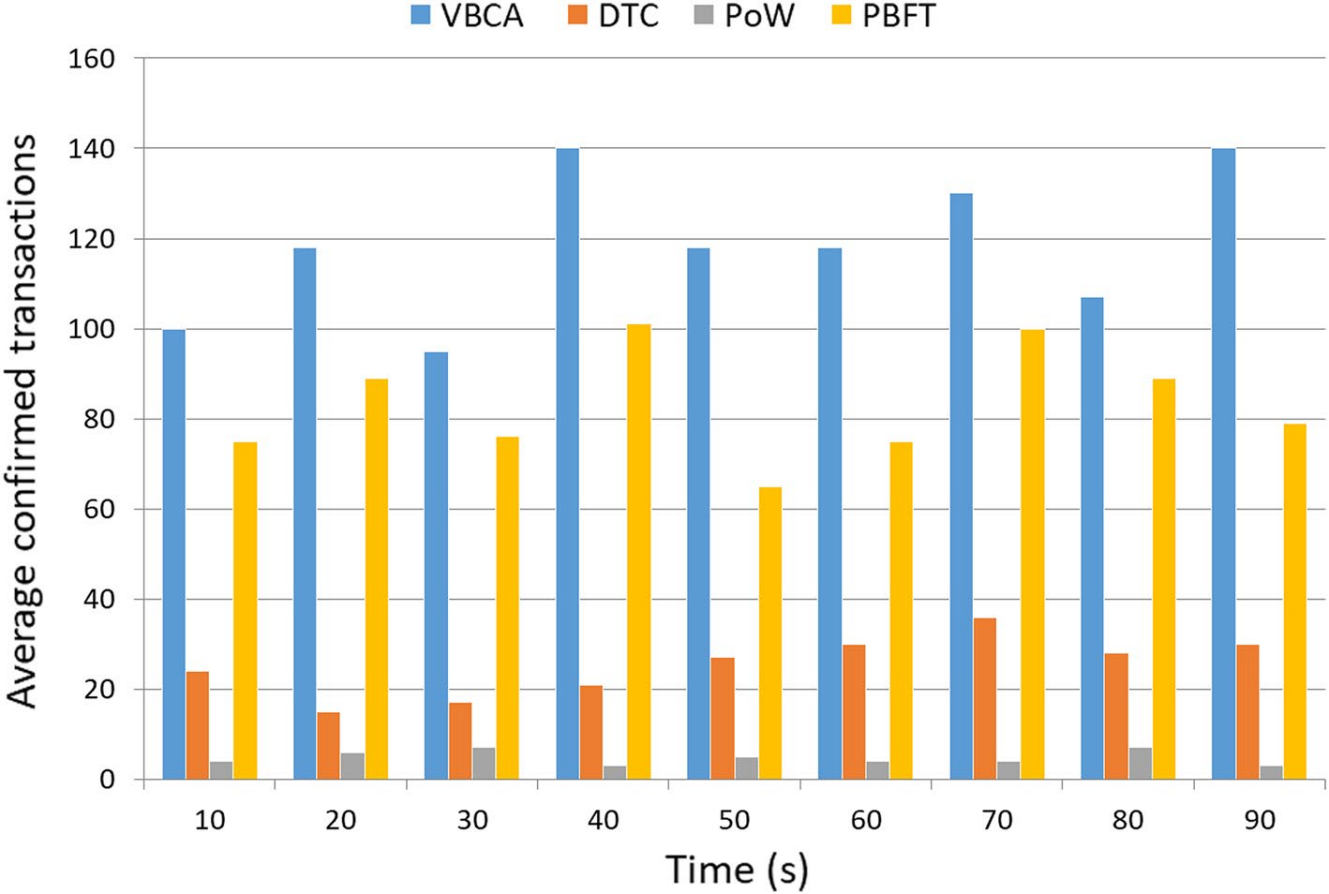
Transactions confirmed per second (throughput).

Figure 4 shows the evaluation results based on throughput. As indicated, the proposed VBCA exhibits better performance than the related schemes. This is due to the leader selection process that reduces the overall transaction confirmation time. When the transaction is confirmed by the leader stationary node, the remaining nodes do not have to wait for the block creation. Messages created per second by vehicular networks need to be confirmed before a specific deadline. However, the PoW scheme only confirms a maximum of 7 transactions per second because of the slow mining algorithm, which negatively impacts the throughput.

### Transaction latency at stationary node

**Figure 5:**
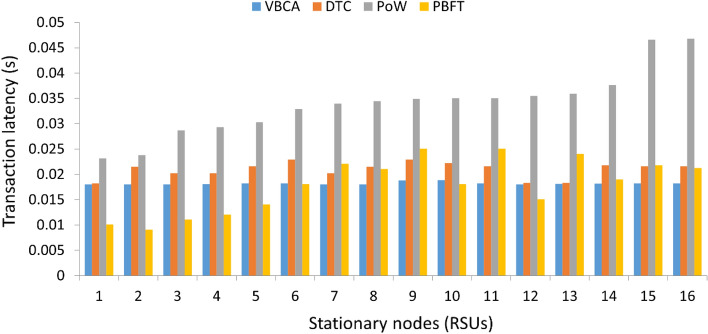
Transaction latency at each RSU.

Figure 5 shows the transaction latency at each node. The proposed VBCA exhibits better performance than the existing schemes, except for the PBFT. The transaction latency heavily depends on the nature of the network. The PBFT is a fully private network, therefore, it has a smaller transaction confirmation time. Alternatively, PoW has greater latency because the transaction has to be confirmed by every peer in the network. We observe that the transaction latency of VBCA at each stationary node is comparatively low because of the consortium blockchain where only the leader stationary node confirms the transaction into the pool.

### Impact of block creation

**Figure 6:**
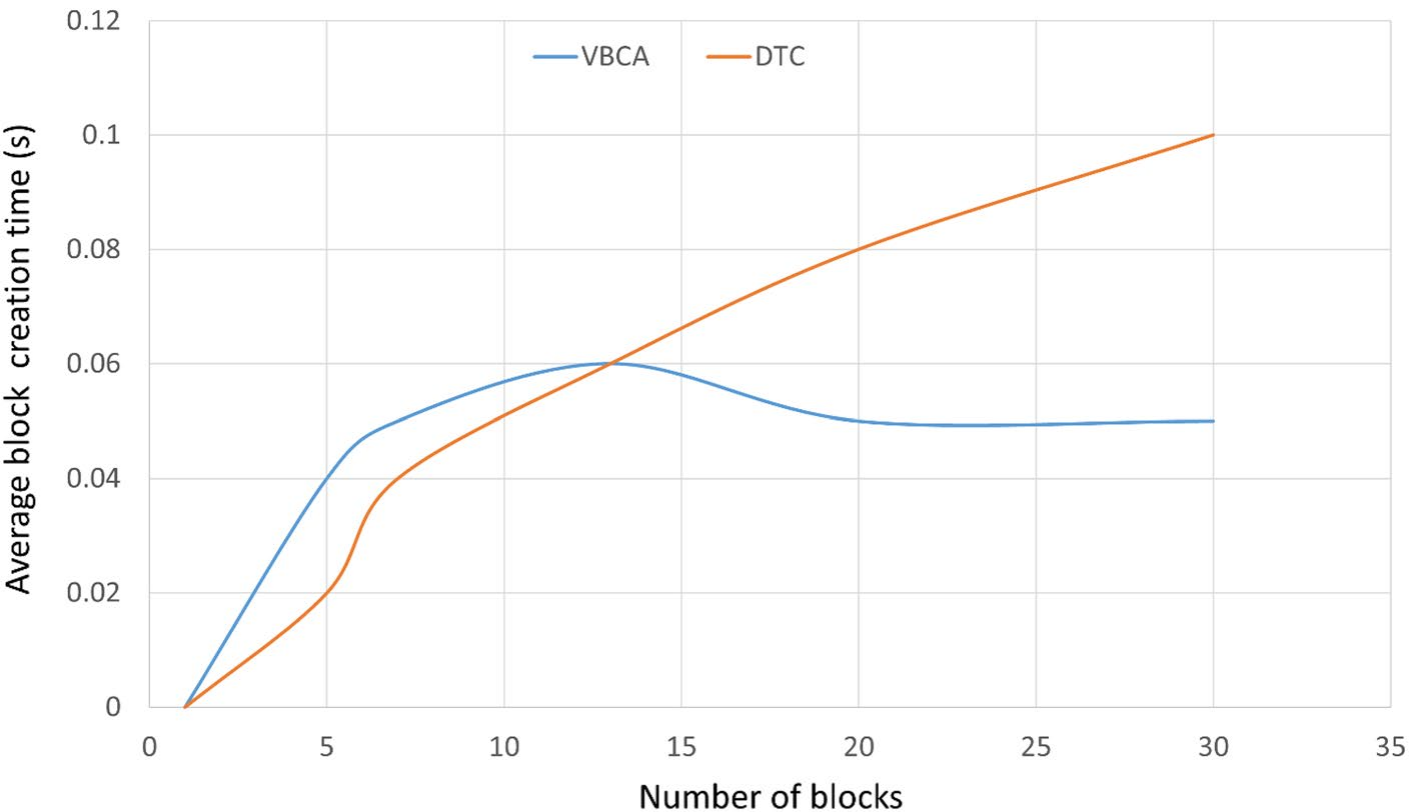
Block creation time.

We set the number of transactions for the genesis block creation to be equal to ten. The genesis block is static and is the first block of a blockchain network. Different leaders create a different number of blocks to ensure the system is decentralized. Each subsequent block constitutes 10 transactions. The proposed VBCA creates the block in the maximum threshold time of 0.05 s. On the contrary, the mechanism used by DTC^42^ initially increases the block creation time for a certain interval until the trusted block creator is selected, after which the block time is fixed at 0.1 s. Figure 6 shows that the block creation time for VBCA becomes constant after almost 20 blocks. This is due to the non-selfish nature of the block creation process in the proposed scheme, where the block must be created within a fixed threshold set for a heartbeat to prevent the selfish mining attack. The block creation times for the remaining schemes contained larger values and are hence not shown in the graph.

### Impact of transaction rate on confirmed transactions

**Figure 7:**
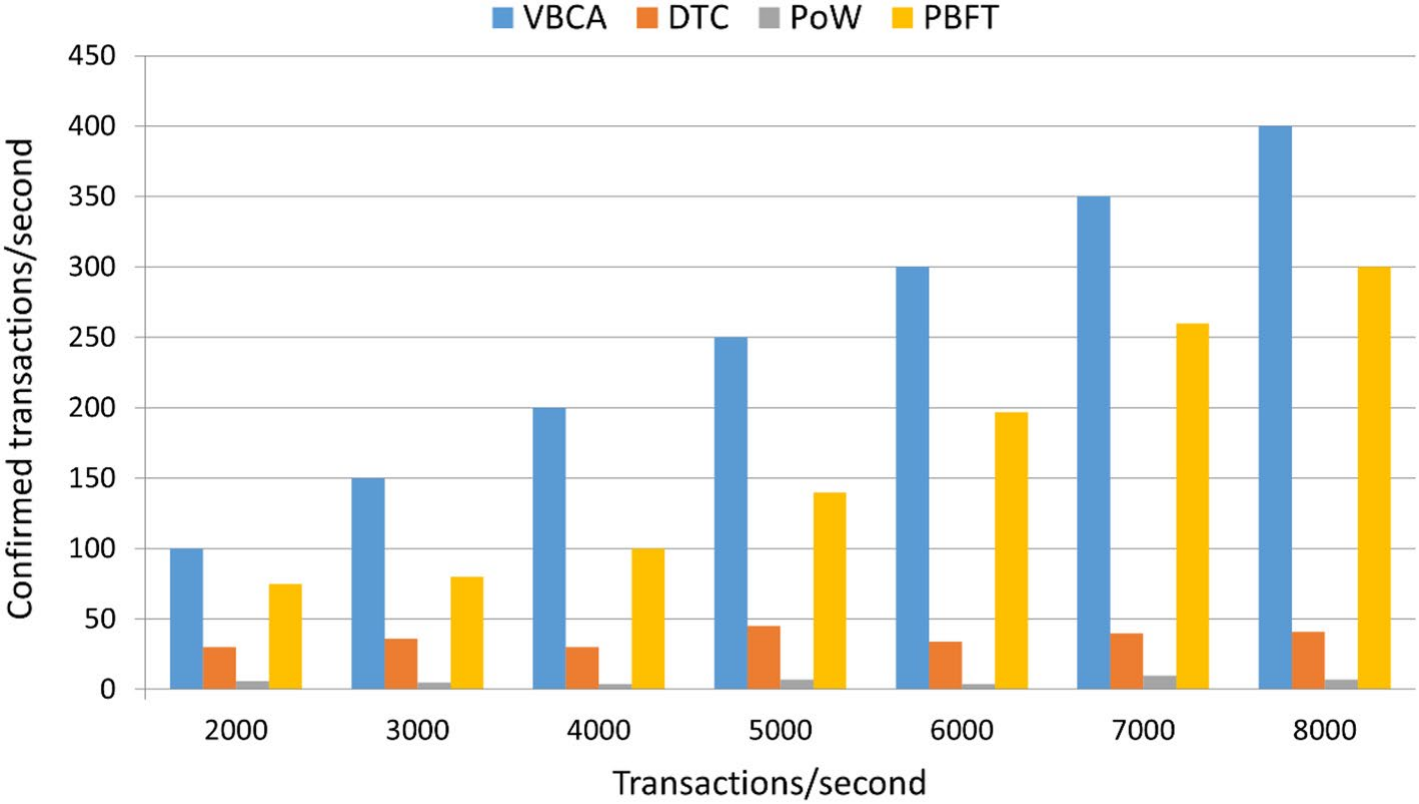
Variation in transaction rate.

We have evaluated different consensus schemes against the transaction rate. The results in Fig. 7 show that VBCA has a higher transaction confirmation rate compared to other approaches. This is due to the Hybrid P2P structure of the proposed scheme where after the validation of the transaction, the respective stationary node forwards the transaction to the leader node, thereby avoiding the long process for validation by every single node in the network^25^.

### Impact of number of blocks per RSU

**Figure 8:**
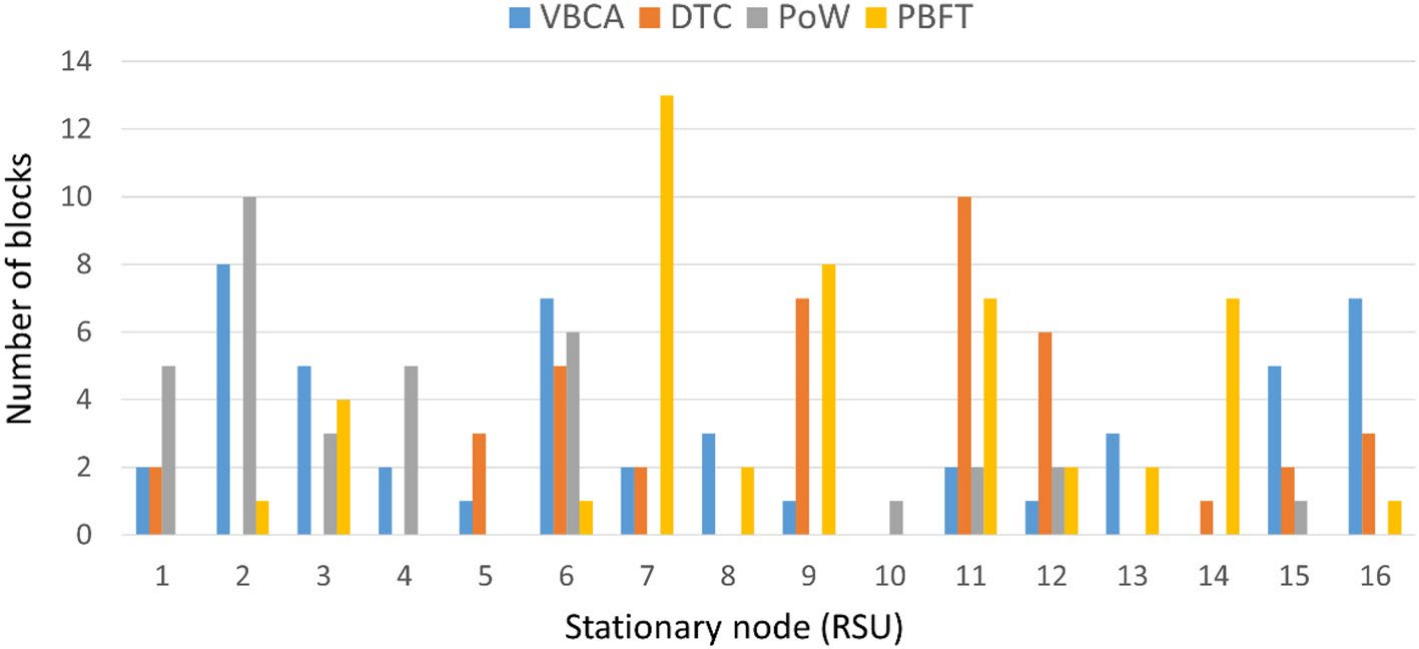
Block creation per node.

The stationary nodes that manage the ledger by appending the latest block to the ledger must be carefully selected to ensure system decentralization. Figure 8 indicates that VBCA performs block creation at almost every node, thus making the system decentralized. On the contrary, PoW^25^ selects the node with greater resources leading to the greater consumption of network resources, centralization, and creating more orphan blocks. Alternatively, the DTC scheme^42^ is trust table-based making it more centralized and exposing the system to a single point of failure due to attacks. Better decentralization in VBCA is achieved by not allowing the consecutive selection of the same leader, thus giving chance to other nodes for block creation.

### Transaction confirmation time for block

**Figure 9:**
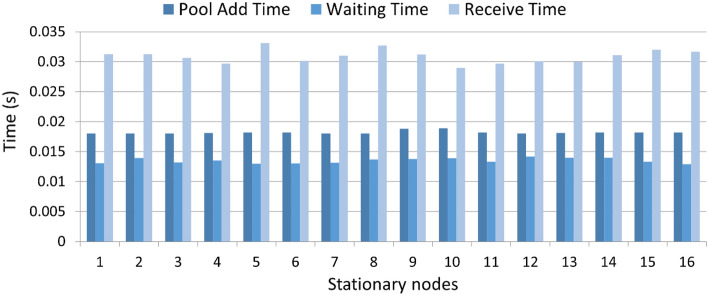
Transaction confirmation for block.

Figure 9 shows the transaction confirmation time per block. The time when the leader confirms the transaction by broadcasting to the network and adding the transaction to the pool is referred to as transaction confirmation time. The transaction confirmation time per block is given as follows:

2 $$ Transaction \;conf.\; time \;per\; block = \mathop \sum \limits_{i} TAdd_{i} + Waitpool\;time_{i} + Ack\;time_{i} . $$

3 $$ Waitpool\;Time = Pick\;timestamp_{i} \left( {Tx} \right) {-} {\text{Re}} cvd\;time\;stamp_{i} \left( {Tx} \right) $$

Here, ${TAdd}_{i}$ is the time when a leader selects the transaction from the pool to add to a block and the block is propagated to other stationary nodes to append into their respective ledgers. Moreover, the parameter ${AckTime}_{i}$ is the time the leader waits until the acknowledgement from each stationary node is received and the block is added.

As we can observe from the results, the existing approaches take more time for transaction confirmation due to their resource-intensive consensus algorithm. Such delays result in an overall increase in network latency and throughput. In addition, the block creation process in the consensus algorithms takes the system towards centralization which violates the essence of blockchain technology. The proposed algorithm separates the transaction confirmation and block generation process to reduce the transaction confirmation latency. The block creation process is also decentralized. Moreover, the proposed algorithm uses a smart contract for the candidate selection to reduce the heartbeat overhead. The transactions are confirmed by the current leader RSU node, which reduces the confirmation time.

## Security analysis

This subsection presents the security performance of the proposed system discussing various types of possible attacks, and how the proposed system can counter those attacks. There are no standardized performance metrics to quantitatively analyze the comparative security performance of vehicular networks using blockchain, as many complex factors are involved including dynamic topology, vehicular speeds, data rates, communication technology, and so on. Therefore, we present a qualitative analysis of security performance of the proposed system highlighting its feasibility in vehicular networks. The proposed system is based on blockchain as a backbone, and therefore inherits all the inherent security features provided by a blockchain network such as, confidentiality, integrity, authentication, nonrepudiation, and immutability.

The following are some of the possible security attacks on the proposed system, and how the system is capable to counter those attacks.

### Integrity violation

- *Attack method:* An attacker tries to modify the contents of any block.
- *Proposed solution:* In the proposed model, the sequence and contents of the blocks are secured using a hash chain where each block has a unique hash value. If an attacker tries to modify the contents of any block, the attacker needs to modify the current block as well as recalculate the hash values of all subsequent blocks in the chain which is extreme resource consuming, thus making it difficult for an adversary to successfully carry out a message modification attack.

### Sybil attack

- *Attack method:* The attacker generates numerous fake identities of a node to spread forged messages in the network^45^. Using such mechanism, an attacker tries to gain the control on the network to carryout illegal activities, such as refusing the transactions.
- *Proposed solution:* The proposed system is resistant to Sybil attacks as it requires a majority of nodes to agree on the validity of a new block before it can be added to the chain. To maintain a strong identify verification process in place, the proposed system maintains an access control list which contains the registered RSUs of the network. The blocks encrypted with the public-key pair and registered in the access control list will be verified by the other RSUs.

### Eclipse attack

- *Attack Method:* An attacker isolates a node from the rest of the network and submits conflicting transactions, potentially causing the node to make decisions based on incorrect information^46^.
- *Proposed solution:* The proposed work ensured that the leaders have robust and diverse communication channels, as well as implemented measures such as peer-review and transaction validation by at least 50% of the RSU nodes.

### Denial of Service (DoS) attack

- *Attack method:* The attacker floods the network with many invalid or malicious transactions, disrupting the normal functioning of the blockchain^47^.
- *Proposed solution:* The proposed system is not vulnerable to DoS attacks because it is not reliant on a single central server or point of failure. Instead, it relies on a network of nodes that all have copies of the blockchain, making it more difficult for an attacker to take down the system by targeting a single point.

### Selfish mining attack

- *Attack method:* Miners try to disrupt the system by withholding newly mined blocks from the network and only releases those when it is to their advantage^48^. This can allow the attackers to earn a disproportionate share of the rewards for mining new blocks at the expense of other miners in the network.
- *Proposed solution:* Our model addresses this issue by requiring all the nodes to agree on the order in which updates are applied to the ledger. If a node mines a block, it needs to propagate the block within the heartbeat timeout, otherwise, it will be unable to reach consensus with the other nodes and the block will not be added to the ledger.

### Transaction ordering dependence attack

- *Attack method:* A transaction ordering attack occurs when a malicious actor attempts to manipulate the order in which transactions are recorded on the blockchain^49^. If the attacker can convince enough of the network nodes to accept its version of the transaction, it may be able to disrupt the normal operation of the network.
- *Proposed solution:* The proposed model applies Last-In-First-Out (LIFO) strategy, where transactions are ordered based on their timestamps such that the transaction with the most recent timestamp is processed first. This can help to prevent the transaction ordering attacks, as it ensures that the order in which transactions are recorded on the blockchain reflects the order in which they were actually made.

## Achievements, limitations, and future research directions

We proposed a blockchain based framework to ensure data security in vehicular network environments. The smart contracts are used to ensure decentralization by letting multiple stationary nodes to append a block in a ledger. The proposed technique demonstrates improvement in the results when compared with the state-of-the-art mechanisms in terms of throughput and the number of blocks created per node while ensuring decentralization. The selfish mining problem is addressed by setting the threshold to create a block in a specified time. Moreover, the transaction latency is also reduced by proposing the separation of transaction confirmation and block creation process.

Like the previous research works, the proposed system also suffers from a few limitations. Cryptography is globally considered as an acceptable security solution for many applications, and our proposed work utilizes cryptography as a first security layer. However, cryptographic based security is still vulnerable to various types of attacks. Especially, the availability of quantum computing devices at adversarial end may increase the security challenges for vehicular networks. Therefore, in future we aim to explore the trust and reputation mechanisms to identify the misbehaving node. Moreover, an incentive mechanism will be introduced to encourage honest nodes in block creation process. Future research will also focus on different vehicular network attacks and their quantitative analysis under different conditions with blockchain implementation.

A factor that can affect the speed of block confirmation is the block size. If the block size is too large, it can take longer for the block to be propagated throughout the network, which can increase the time it takes to confirm the block. On the other hand, if the block size is too small, it may not be able to accommodate enough transactions, which can lead to delays in processing transactions. To address this issue, in future we aim to implement dynamic block sizing, which allows the block size to adapt to the current transaction rate and network conditions. This can help in reducing the block propagation delay and speed up the block confirmation process.

Separating beacon messages from event messages can also help in improving the efficiency of the blockchain network. Beacon messages are used to synchronize the state of the network and to ensure that all nodes have the same view of the blockchain. Event messages, on the other hand, contain information about transactions and other events that have occurred on the network. By separating these two types of messages, it can be easier to process and disseminate event messages, which can help avoid delays in the network.

Scalability is another limitation of the proposed system as the number of vehicles can be considerably large in a real-word scenario. We aim to use sharding in the future model which will help us improve the scalability performance, security, and resource requirements of a vehicular network by dividing the network into smaller, independent units called shards. Each shard can process transactions and maintain its own copy of the blockchain, allowing the overall network to handle larger volumes of transactions without sacrificing performance and making it more difficult for a single entity to gain control or launch an attack. Additionally, sharding can reduce the resource requirements for each RSU or vehicle by allowing them to process transactions and maintain their own copy of the blockchain without having to store and process the entire network's data.

## Conclusion

The paper presents a blockchain based data security solution for vehicular networks. The integration of blockchain technology in the vehicular networks has the potential to transform the transportation system and introducing many applications that can build on top of blockchain. There is a tradeoff between providing security and timely message dissemination in vehicular networks based on blockchain. If the consensus mechanism is based on utilizing huge resources of the miner such as time, computational power, storage, and coins, stronger will be the security of the blockchain network. However, increasing the security can result in a decrease in throughput. It is also noted that the increase in the number of transactions in the block can lead to a delay in block creation and propagation time. It has been observed that by increasing the number of generated transactions by vehicles, there is also an increase in the confirmed transactions.

## Data availabilty

The authors did not use any external dataset for simulation. The simulations are performed using NS-3 that has built-in modules to generate synthetic mobility and connectivity patterns.
